# Measured and forecasted weather and power dataset for management of an island and grid-connected microgrid

**DOI:** 10.1016/j.dib.2021.107513

**Published:** 2021-10-28

**Authors:** Danilo P. e Silva, José L. Félix Salles, Jussara F. Fardin, Maxsuel M. Rocha Pereira, Vinícius C. Ottz, Flavio B.B. da Silva, Eduardo G. Pignaton

**Affiliations:** a Federal Institute of Education, Science and Technology of Espírito Santo (IFES), Serra, E.S., Brazil; b Electrical Eng. Department, Federal University of Espírito Santo (UFES), Vitória, E.S., Brazil; c Industrial Technology Department, Federal University of Espírito Santo, Vitória, E.S., Brazil; d Professional Master Program in Control and Automation Engineering of the Federal Institute of Education, Science, and Technology of Espirito Santo, Serra, E.S., Brazil

**Keywords:** Measured weather data, Forecasting weather data, WRF, Renewable energy sources, Microgrid management

## Abstract

This article presents the weather and power data files from renewable sources used to solve the economic dispatch problem of a microgrid that operates in the isolated and grid-connected modes. Methodology is used in the research article “Management of an island and grid-connected microgrid using hybrid economic model predictive control with weather data” (Silva et al., 2020). Automatic stations located in the Brazil's south and northeast furnished the weather data (global horizontal irradiance, temperature, and wind speed). A script generates files containing weather forecasts from one-day ahead using the geographical coordinates of the weather stations. Hybrid models, characterized by real and binary variables, use the weather forecasting data to calculate the photovoltaic and wind power forecasts. A microgrid management algorithm uses these forecasts to solve the optimal economic dispatch problem. This data-in-brief paper presents five datasets for each weather station: (i) Weather dataset downloaded from the website of the National Meteorological Institute, (ii) Weather research and forecasting (WRF) dataset derived from the raw data generated by the weather research and forecasting model, (iii) Weather dataset that joins the forecasted data with the measured data in a single file, (iv) Handled dataset that treats some gaps in the weather dataset and converts it to other formats, (v) Files containing only the temperature, global horizontal irradiance, and wind speed data, (vi) Files containing the measured and forecasted wind and solar power.

## Specifications Table

**Table:** 

Subject	Energy engineering and power technology.
Specific subject area	Microgrid management. Weather forecasting model.
Type of data	Temperature, global horizontal irradiance (GHI) and wind speed measured/forecasted dataset of Natal (.mat and .dat formats); temperature, irradiance and wind speed measured/WRF dataset of Santa Vitoria do Palmar (.mat and .dat formats); WRF dataset of Natal (xlsx); WRF dataset of Santa Vitoria do Palmar (xlsx); weather dataset of Natal (xlsx) and weather dataset of Santa Vitoria do Palmar (xlsx); weather measured dataset of Natal (.csv); weather measured dataset of Santa Vitoria do Palmar(.csv); photovoltaic (PV) and wind power dataset of Natal (.mat and .dat formats); photovoltaic and wind power dataset of Santa Vitoria do Palmar (.mat and .dat formats).
How data were acquired	The measured weather data were extracted from the National Meteorological Institute (INMET) [2] located in Brazil. The forecasted meteorological data were obtained through the global WRF weather research and forecasting model [3].
Data format	Raw, converted, processed and filtered data.
Parameters for data collection	All data were collected at Natal and Santa Vitoria do Palmar weather stations located in Brazil. Data were collected between 01/08/2020 to 01/21/2021 for Natal and from 02/15/2020 to 01/21/2021 for Santa Vitória do Palmar. The measured data have a one-hour-sample rate and the forecasted data have a 10 m sample rate.
Description of data collection	The measured data were obtained from the INMET website [2] through the historical data download of the Natal and Santa Vitoria do Palmar automatic stations, respectively. The forecasted data were generated daily by scripts of the WRF model and configured for the geographical coordinates of the automatic stations of Natal, and Santa Vitoria do Palmar.
Data source location	Institution: INMET City/Town/Region: Natal/RN Country: Brazil Latitude: –5.837187° Longitude: –35.207921° Institution: INMET City/Town/Region: Santa Vitoria do Palmar/RS Country: Brazil Latitude: –33.742297° Longitude: –53.372218° Primary data sources: • daily WRF forecast spreadsheets for Natal(.dat). • daily WRF forecast spreadsheets for Santa Vitoria do Palmar(.dat). • weather measured dataset of Natal (.csv). • weather measured dataset of Santa Vitoria do Palmar (.csv).
Data accessibility	All the weather datasets, power datasets and the transparency document associated with this article are available at our Mendeley Data repository. http://dx.doi.org/10.17632/skxgmkc64k.4The source codes is available on GitHub and has been archived in the Zenodo open-access repository.Weather dataset code: https://doi.org/10.5281/zenodo.5590840Power dataset code: https://doi.org/10.5281/zenodo.5590439
Related research article	Danilo P Silva, José L. F. Salles, Jussara F. Fardin, Maxsuel R. Pereira, Management of an island and grid-connected microgrid using hybrid economic model predictive control with weather data. Applied Energy. https://doi.org/10.1016/j.apenergy.2020.115581

## Value of the Data

• Weather data is important to calculate the measured and forecasted power of renewable sources through mathematical models. These models convert the temperature, GHI and wind speed data into photovoltaic and wind powers. The management algorithm presented in [1] use all these data to solve the optimal economic dispatch problem.
• The data presented in this brief-data article can assist researchers and companies in the energy sector to simulate and compare various energy management and conversion systems that combine heat and power [4], intelligent buildings [5], virtual power plants [6], microgrids [7], among others.
• These meteorological data can validate energy conversion models and the management algorithm of renewable energy based on forecasts [8]. The power data help researchers to develop power forecasting techniques and mathematical models that convert weather data into solar and wind power.
• The data presented here allows energy management research to produce real results and more reliable renewable energy forecast to consumers. For example, in [9], the atmospheric air inlet temperature of the solid oxide fuel cell model is considered constant. However, according to [8], more realistic results should be obtained in [9] by considering real and forecasted ambient temperature values.

## 1 Data Description

The shared data are essential to obtain wind and solar power from any power systems that include these renewable energy generations, such as microgrids, virtual power plants, heat and power combined systems, wind farms, and solar energy farms. The paper [1] studied a microgrid characterized by photovoltaic panels, wind turbines, converters (photovoltaic and wind), load control panel, controllable resistive loads, battery bank type-energy storage system, and standard connection panel. During the initialization of the optimization algorithm proposed by [1], it is necessary to define the planning (Np) and forecasting (N) horizons. The planning horizon defines the period of time needed to manage the microgrid and also the amount of meteorological data forecasted by the WRF model at the end of the previous day. These forecasted meteorological data are used by hybrid models to calculate the photovoltaic and wind power forecasts from the current sample k until k+N. The photovoltaic power depends on the ambient temperature and the GHI. The wind power, on the other hand, depends on the wind speed and its direction. The forecasts of photovoltaic and wind powers contained in the actual and future samples (k,k+1,k+2,...k+N−1), previously determined by hybrid models, must be sent to the optimization algorithm at the current time kTs, where Ts is the sample time. Next, the optimization algorithm manages in real time the status of the battery bank and performs the grid connection/disconnection of the microgrid. This procedure is repeated from instant (k+1)Ts until the total planning period NpTs. An important note is that the size of the forecast dataset must be less than or equal to k+N. Otherwise, the algorithm will report an error at the end of its execution due to lack of forecasting data. The measured and forecasted meteorological data allow performing the following studies:

• Determine the forecasting errors of the meteorological data of temperature, GHI, and wind speed.
• Determine the forecasting errors of the wind and photovoltaic powers.
• Perform the microgrid management strategy's sensitivity analysis in relation to forecast errors, comparing power generation forecasts and their respective actual data.

**Fig. 1:**
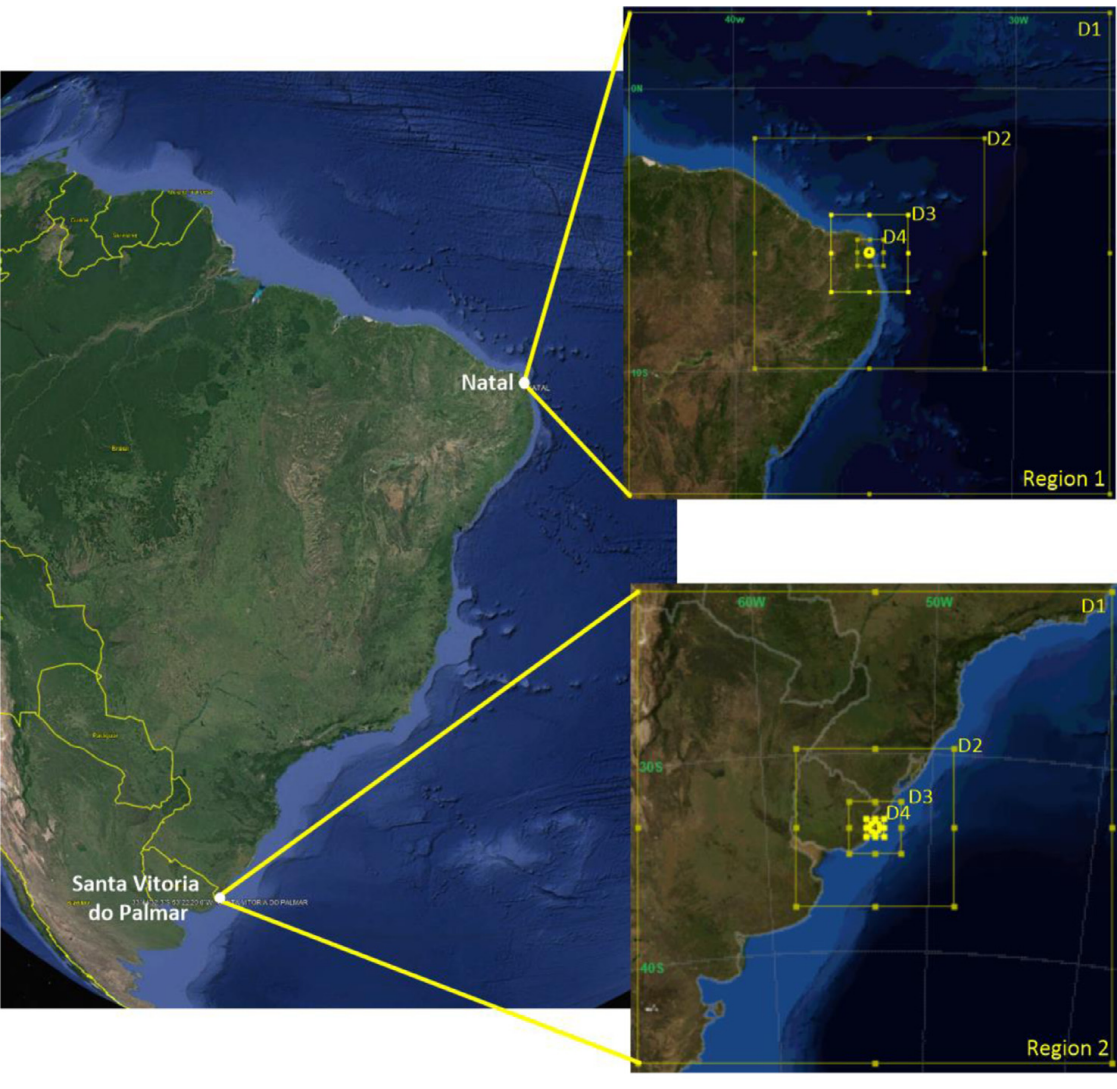
Domain configuration of WRF model centered in Natal/RN (Region 1) and Santa Vitoria do Palmar/RS (Region 2).

In this study, the Weather Research and Forecasting model (WRF version 4.1.2, available at https://www2.mmm.ucar.edu/wrf/users/) [3] was used to simulate two distinct regions (see Fig. 1), each one using the geographic distribution in Lambert projection of the four nested domains (D1, D2, D3 and D4). The region 1, centered in Natal-RN, is in the Northeast of Brazil and region 2, centered in Santa Vitória do Palmar-RS, is in the South of Brazil. Both include the Western portion of the South Atlantic Ocean and have the same configuration domain, with horizontal grid resolutions of 27 km (D1; mesh size of 70 × 70), 9 km (D2; mesh size of 100 × 100), 3 km (D3; mesh size of 100 × 100), and 1 km (D4; mesh size of 100 × 100). The simulations were performed using 31 vertical levels for each domain, which 16 are located within the Planetary Boundary Layer (PBL), with a top set at 50 hPa. The model outputs had a temporal resolution of 10 min. Initial boundary conditions were obtained from the National Centers for Environmental Prediction (NCEP). Daily data has time resolution every 3 h from the Global Forecast System (GFS) [10] available at https://ftp.ncep.noaa.gov/data/nccf/com/gfs/prod.

Furthermore, to minimize possible instability problems in the simulation, a spin-up cycle was added, where the first 3 h of each prediction cycle were not included in the analyzes for a 24 h period of simulation.

The locations may be briefly described as follows:

1. Natal-RN. Capital of the State of Rio Grande do Norte, was founded in 1599 on the banks of the River Potengi. It is known for its natural beauty, beautiful beaches, dunes, lagoons and coconut trees. Natal has a tropical rainy climate with dry summer, and relatively low temperature ranges and relatively high air humidity, due to its location on the coast. The state capital boasts the title of City of the Sun due to its high solar luminosity that exceeds 2900 h per year. The annual rainfall index exceeds 1700 millimeters (mm), concentrated between the months of March and July. Stronger winds happen especially between the months of August and October.
2. Santa Vitoria do Palmar-RS, Brazil. Located in the extreme south of Brazil, it borders on the north with the city of Rio Grande and on the south with the city of Chuí, also bordering Uruguay. The eastern part of Santa Vitória do Palmar is bathed by the Atlantic Ocean, totaling 140 km of coastline with 4 maritime lighthouses installed. Between the sea and the BR 471, there is the Mangueira lagoon, which was formed by the rise and fall of the sea over thousands of years. It is the only lagoon in Brazil that has the Spirulina microalgae capable of absorbing pollutants from the atmosphere. The city's climate is subtropical, with moderate summers and cool winters. The annual average maximum temperature is around 22°C and the minimum around 12 °C. It has an average wind speed of over 6.5 m/s, which is considered a good average for installing wind turbines. In general, the rains are well distributed throughout the year, with a little more frequency in winter.

Table 1 shows the main physical options selected. More information on these physical options can be found in [3].

**Table 1:** The main physical options selected to WRF.

Data	NCEO GFS
Data interval	3 h
Pressure at model top	50 hPa
Mesh size	D1: (70 × 70) x 31 D2: (100 × 100) x 31 D3: (100 × 100) x 31 D4: (100 × 100) x 31
Grid resolution	D1: (27 × 27) km D2: (9 × 9) km D3: (3 × 3) km D4: (1 × 1) km
Map projection	Lambert
Center Lat/Lon	Region 1 (Natal-RN): 5°50′13.87′′S, 35°12′28.52′′W Region 2 (Santa Vitória do Palmar-RS): 33°44′32.27′′S, 53°22′19.98′′W
Model dynamics	Non hydrostatic
Integration time step	60 s
Vertical coordinates	Terrain-following sigma co-ordinate system with 31 vertical levels
PBL scheme	Yonsei University sheme (YSU)
Surface layer option	Monin-Obukhov similarity theory (MM5 MRF PBL)
Cumulus parameterization option	Betts-Miller-Janjic
Cloud microphysics	Kain-Fritsch
Longwave radiation	RRTM scheme
Shortwave radiation	MM5 scheme
Landuse	MODIS - including lake category

The code read_wrf_nc.f, available in https://www2.mmm.ucar.edu/wrf/src/read_wrf_nc.f, was used to read output files of the WRF in the exact center points in D4 domain to both locals, Natal-RN and Santa Vitoria do Palmar-RS. The WRF model was used for prognostic of air temperature, global horizontal irradiation (GHI), wind speed, and estimations of the photovoltaic power. Focusing on the model central domain with 1 km resolution, the difference due to the variation in the resolution of the WRF data and the INMET data is showed in the Table 2 We can observe that Santa Vitória do Palmar has an average temperature 37% lower than Natal, but MAE and MAPE were higher in relation to these values for Natal. In the case of GHI, the WRF model performed very well in Santa Vitória do Palmar with low MAE and MAPE values. Natal has an average GHI 22% higher than Santa Vitória do Palmar and also had higher MAE and MAPE values. In terms of wind speed, Santa Vitória has an average speed 36% higher than Natal, with MAE and MAPE values also higher than Natal values. Thus, the photovoltaic generation in Natal is 21% greater than the photovoltaic generation in Santa Vitória do Palmar. On the other hand, wind generation in Santa Vitória do Palmar is 129% greater than wind generation in Natal. The MAPE values for power are greater than the MAPE values for the meteorological data due to the additional error arising from the mathematical models for the calculation of photovoltaic and wind powers.

**Table 2:** Mean absolute error (MAE) and mean relative error (MAPE) between simulation WRF and real INMET data.

	*Variable*	*Average WRF*	*Average INMET*	*MAE*	*MAPE (%)*
*Natal-RN*	Temperature	27.10°C	26.58°C	0.52°C	1.96
	GHI	288.8 W/m²	251.31 W/m²	37.49 W/m²	14.92
	Wind Speed	4.3 m/s	3.78 m/s	0.52 m/s	13.75
	PV power	425.4 W	349.6 W	75.8 W	21.68
	Wind power	192.9 W	142.0 W	50.9 W	35.81

*Santa Vitoria do Palmar-RS*	Temperature	15.70°C	16.62°C	0.92°C	5.53
	GHI	205.3 W/m²	205.0 W/m²	0.3 W/m²	0.15
	Wind Speed	6.10 m/s	5.16 m/s	0.94 m/s	18.21
	PV power	298.4 W	288.8 W	9.6 W	3.24
	Wind power	438.0 W	325.7 W	112.3	34.47

**Fig. 2:**
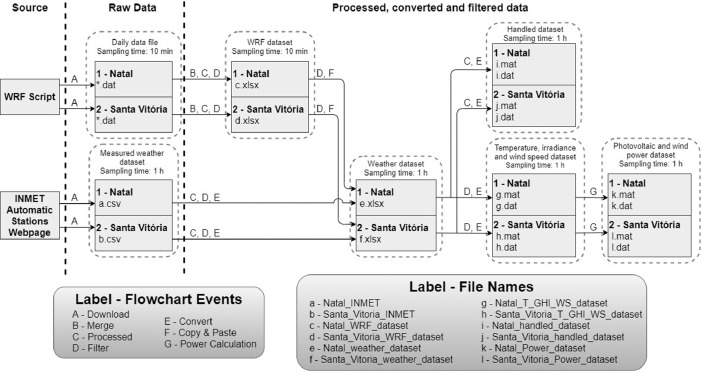
Flowchart for obtaining and processing the meteorological and power dataset.

The Fig. 2 shows the flow chart to obtain the data files. The weather data of GHI, temperature, and wind speed were extracted from INMET [2]. We used the weather research and forecasting (WRF) model [3] for estimations of the direction and speed of wind, air temperature, and global radiation.

The raw data obtained through INMET and the WRF model are processed and filtered into five datasets:

1. **WRF dataset**. It has data related to the calculation of wind speed and direction at 10 m height, the conversion of Coordinated Universal Time (UTC) to the local time zone of the microgrid, and the resampling of the meteorological data of Natal and Santa Vitória.
2. **Weather dataset**. It has measured data from the automatic weather stations and their respective forecasted temperature, GHI, and wind speed, obtained through the WRF model.
3. **Handled dataset:** It consists the conversion of weather dataset to .mat/.dat files and filling the gaps of measure data.
4. **Temperature, GHI, and wind speed dataset**. It has data related to the graphs and tables presented in the article's numerical result section [1] and the power data presented in the next item.
5. **Photovoltaic and wind power dataset**. It has the actual and forecasted power data obtained from the mathematical models proposed by [1] and the temperature, GHI, and wind speed dataset.

The following is a detailed description of each data file available in [11].

### 1.1 Raw data

#### 1.1.1 Daily dataset

It has daily raw files (.dat) with the one-day ahead weather forecasts of the data described in Table 3 for coordinates (X, Y, Z).

**Table 3:** Variables presented in the raw daily WRF dataset.

Variables	Description
Time	Date and UTC time
X	INDEX IN X (WEST-EAST) DIRECTION
Y	INDEX IN Y (SOUTH-NORTH)
Z	INDEX IN Z (VERTICAL) DIRECTION
P	PERTURBATION PRESSURE
PB	BASE STATE PRESSURE
PH	PERTURBATION GEOPOTENTIAL
PHB	BASE-STATE GEOPOTENTIAL
U	X-WIND COMPONENT
V	Y-WIND COMPONENT
W	Z-WIND COMPONENT
HGT	TERRAIN HEIGHT
U10	U AT 10 m (X-WIND COMPONENT AT 10 m)
V10	V AT 10 m (Y-WIND COMPONENT AT 10 m)
T2 (K)	TEMPERATURE IN KELVIN AT 2M
SWDOWN	SHORTWAVE IRRADIATION^1^

The variables wind speed, wind direction, air temperature and GHI was forecasted in the same heights of the measurement instruments of automatic stations. The data were collected between from 01/08/2020 to 01/21/2021 for Natal and from 02/15/2020 to 01/21/2021 for Santa Vitória do Palmar, and they were sampled every 10 min. The WRF model generated 720 daily files (379 files for Natal and 341 files for Santa Vitoria do Palmar). These raw data are sequentially organized in the WRF dataset files.

#### 1.1.2 Measured weather dataset

It has raw files (.csv) generated by INMET automatic stations collected from 01/08/2020 until 01/21/2021 for Natal and from 02/15/2020 until 01/21/2021 for Santa Vitória do Palmar. The field names of raw files are in Portuguese. Table 4 presents the translation of each field of .csv files. Table 5 summarizes the measurement instruments specifications and heights of surface. The weather station measures at every minute the meteorological information such as temperature, humidity, atmospheric pressure, precipitation, wind speed and direction, solar radiation. Every hour, these data are available to be transmitted, via satellite or cell phone, to INMET's headquarters in Brasília. The received data are validated through quality control and stored in a dataset. The data are also available free of charge in real-time, through the internet [2] to elaborate on various meteorological services such as meteorological reports, weather surveillance, and weather forecasts and patterns. These data are useful for many applications, such as hydrology and oceanography research [12].

**Table 4:** Translation of INMET variables.

Variables	Translation
Data	Date
Hora (UTC)	UTC hour
Temp. Ins. (C)	Temperature Inst
Temp. Max. (C)	Temperature Max
Temp. Min. (C)	Temperature Min
Umi. Ins. (%)	Humidity Inst
Umi. Max. (%)	Humidity Max
Umi. Min. (%)	Humidity Min
Pto Orvalho Ins. (C)	Dew temperature Inst
Pto Orvalho Max. (C)	Dew temperature Max
Pto Orvalho Min. (C)	Dew temperature Min
Pressao Ins. (hPa)	Pressure Inst (hPa)
Pressao Max. (hPa)	Pressure Max (hPa)
Pressao Min. (hPa)	Pressure Min (hPa)
Vel. Vento (m/s)	Wind speed (m/s)
Dir. Vento (m/s)^2^	Wind direction (°)
Raj. Vento (m/s)	Max speed (m/s)
Radiacao (KJ/m^2^)	Short wave radiance
Chuva (mm)	Precipitation (mm)

**Table 5:** Measurement instruments of the Natal and Santa Vitoria do Palmar automatic stations. Courtesy of INMET-Brazil [2].

Num	Parameters	Sensor	Manufacturer	Operation principle	Unit	Height of Sensors (m)
1	Air Temperature	Thermometer	Vaisala	It is the measurement of a platinum resistance using a 1 mA excitation current and voltage measurements across a Pt100 element and a 100-ohm reference resistor.	°C	2,0
2	Humidity	Hygrometer	Vaisala	It is a measure of the capacitance of a thin capacitive polymer film, i.e., using the HUMICAP180 sensor.	%	2,0
3	Global Horizontal Irradiance	Pyranometer	Kipp & Zonen	The measure of radiant energy that is absorbed by a black-and-white-painted disk under protective glass domes.	kJ/m²	1,5
4	Atmosferic Pressure	Barometer	Vaisala	It is a capacitance measurement based on an RC (Resistance-Capacitance) oscillator circuit with a BAROCAP sensor, three reference capacitors and a capacitive temperature sensor.	hPa	1,5
5	Precipitation	Pluviometer	Vaisala	The sensor operates on the tipping-collector principle, and emits an output pulse from the closing of the circuit each time a certain amount of water is collected.	mm	1,5
6	Wind direction	Wind vane	Vaisala	It is a weathervane that produces a 6-bit code. Infrared LEDs and phototransistors are mounted on six beams on each side of a code disc that determines the wind direction.	Degrees	10,0
7	Wind speed	Anemometer	Vaisala	It consists of a rotating shell anemometer that uses an optical-electronic chopper (switch) circuit to determine the rotation speed of the shell axis.	m/s	10,0
8	Wind direction and speed	Sonic	Gil Instruments	It is an ultrasound pulse that it takes to travel from the North transducer to the South transducer, and compares it to the time it takes for a pulse to travel from the South transducer to the North transducer. The same time comparison is performed between the Western transducer and the Eastern transducer.	Degrees and m/s	10,0

### 1.2 Processed, converted and filtered data

#### 1.2.1 WRF dataset

This dataset contains the one-day-ahead forecasts of the Natal and Santa Vitória daily weather data. In addition to the meteorological data described in Table 3, this spreadsheet includes:

• Additional columns with local time (TIME (LT= LOCAL TIME), the temperature in °C (T2(°C)), calculation of the resulting wind speed (Wspeed) at 10 m height, which corresponds to the automatic station's height and the estimation of the wind direction in degrees (Dir (°)).
• Additional columns with the variables U10, V10, Wspeed, Dir., SWDOWN, and T2 (°C) sampled at each hour.

#### 1.2.2 Weather dataset

It has spreadsheets that contain all the data from the measured weather dataset and the forecast of temperature, GHI, wind speed and direction speed. The variables are described in Table 6.

**Table 6:** Variables presented in the weather dataset.

Variables		Description
Date		

Hour	UTC	UTC hour
	Real	Local hour (Brasilia Hour)

Temperature (°C)	Inst	Instantaneous temperature
	Máx	Maximum temperature
	Mín	Minimum temperature
	Forecasted (WRF)	Forecasted temperature obtained by WRF simulations

Humidity (%)	Inst	Instantaneous humidity
	Máx	Maximum humidity
	Mín	Minimum humidity

Dew temperature (°C)	Inst	Instantaneous dew temperature
	Máx	Maximum dew temperature
	Mín	Minimum dew temperature

Pressure (hPa)	Inst	Instantaneous pressure
	Máx	Maximum pressure
	Mín	Minimum pressure

Wind	Speed (m/s)	Wind speed in m/s
	Direction (°)	Wind direction in degrees
	Forecasted speed (WRF)	Forecasted wind speed obtained by WRF simulations
	Forecasted direction (WRF)	Forecasted wind direction obtained by WRF simulations
	Max speed (m/s)	Maximum registered wind speed

Short wave radiance	(kJ/m²)	Short wave radiance in kJ/m²
	(W/m²)	Short wave radiance in W/m²
	Forecasted (W/m²)	Forecasted short wave radiance obtained by WRF simulations

Precipitation	(mm)	Instantaneous precipitation

In this dataset there are some gaps of the measured data obtained from INMET in the months of October 2020 to January 2021. It is up to the user to choose the best way to treat these gaps.

#### 1.2.3 Handled dataset

The measured weather data contained in the weather dataset had gaps for some days in the months of October 2020 to January 2021. If the user wants to work with the complete dataset, the weather dataset was converted from .xls to .mat/dat. This conversion made in MATLAB includes filling these gaps with the average of the measured values of each time. These new datasets do not have column 1 of the weather dataset as MATLAB was not able to read and convert the weather dataset's date format. The code used to simulate the handlet dataset is available at https://github.com/danilopsv/Weather-dataset-code.git.

#### 1.2.4 Temperature, **GHI** and wind speed dataset

The files in this dataset have the filtering variables of the weather dataset. This procedure is necessary because the conversion to the .mat/.dat extension of the complete meteorological dataset contains information that is not used by the optimization algorithm. This generates a high and unnecessary computational cost. The temperature and GHI are used to calculate the photovoltaic power generation and the wind speed is used to calculate the power generation of the wind turbine. See Table 5 to check the heights of these variables. Columns 1, 3, and 5 refer to the measured data of temperature, GHI, and wind speed, respectively. Columns 2, 4, and 6 are the temperature, GHI, and wind speed forecasting data, respectively. The sample rate is one hour. The code used to simulate the temperature, GHI and wind speed dataset is available on https://github.com/danilopsv/Weather-dataset-code.git and Zenodo [13]..

#### 1.2.5 Photovoltaic and wind power dataset

The mathematical models of the wind system and the photovoltaic system use real equipment specifications. The photovoltaic system consists of six panels of 250Wp each connected in series and an 2000W inverter. The wind system consists of a 1000W wind turbine 2.46 meters in blade diameter, and an 1500W inverter. In addition to the technical specifications of the equipment, the mathematical models of solar and wind generation in [1] calculated the power data, in Watts, through the temperature, GHI, and wind speed dataset, starting from 01/08/2020 to 01/20/2021 for Natal, and from 02/15/2020 to 01/20/2021 for Santa Vitória do Palmar. Columns 1 and 2 refer to the measured and forecasted wind power, respectively. Columns 3 and 4 represent the measured and forecasted photovoltaic power, respectively. The code used to simulate the power dataset is available on https://github.com/danilopsv/Power-dataset-code.git and Zenodo [14].

## 2 Experimental Design, Materials and Methods

To generate the meteorological data files, we follow the following procedure (see Fig. 1):

A - Download via webpage or file generation through a script.

On the INMET automatic stations website, we choose the location, the start date, and the end date of the data. The INMET website generates the data and a download link in .csv format. The global WRF model consists of scripts defined by location chosen through geographic coordinates. We specify in the scripts which weather variable will be forecasted, the forecasting time horizon, and the sampling rate. These scripts generate daily .dat files with a 10 min sample rate.

B - Merging files.

The WRF generated daily forecasting data in several files. It was necessary to join this data set in a single file to represent a time interval larger than one day. In this case, the data was merged in a single spreadsheet as follows:

• Natal: from 01/08/2020 at midnight to 01/21/2021.
• Santa Vitória do Palmar: from 02/15/2020 at midnight to 01/21/2021.

C - Data processing.

It performs the following tasks:

• Convert the temperature given in Kelvin to degree Celsius and the supplied solar radiation in $kJ/(h.m^{2})$ to GHI in W/m² considering that $1.0\;W/m^{2}=3.6\;kJ/h.m^{2}$.
• Calculate the resulting wind speed and direction at 10 m height using the following equations: $Wspeed=\;\sqrt{{U_{10}}^{2}+{V_{10}}^{2}}\;$and, $dir=mod(\left(\frac{180^{\circ }}{\pi }\right)\times \;\text{arctan}2\left(V_{10},U_{10}\right)+180^{\circ },\;360^{\circ })$, where U_10_ and V_10_ are the wind speed components X and Y, respectively.
• Replace the outlier data of GHI due to the inconsistencies with their respective predicted values.

D – Data filtering.

Not all raw data was relevant to the microgrid management simulation in [1]. Only the temperature, GHI, and wind speed data are necessary. We perform the filtering of these data from the MS Excel filter command.

E – File conversion.

It converts the raw data to a more user-friendly format like the .xlsx extension. Therefore, it was possible to develop mathematical equations and data filtering directly in the spreadsheet. Besides, it was possible to convert the files to .mat format, an extension used in MATLAB to simulate the microgrid optimization algorithm presented in [1], and .dat format as an additional option for the user. This last conversion includes to fill the gaps of the measured data with the average of the values of each hour. Suppose the average measured temperature of Natal-RN at 2:00 A.M. and 6:00 A.M. are 24 °C and 27 °C respectively. The data processing script fills the gaps with these averages at the respective hours of days when the measured temperature is not available.

F – Copy and paste data.

The WRF forecasting data was copied and pasted into the same data file that contains the weather data. The unification of these data facilitates the extraction and conversion of the data predicted and measured by simulation software such as MATLAB and it assists in the graphical comparison between the measured and forecasted data.

G – Power calculation

Wind and photovoltaic power generations were calculated through mathematical models developed in MATLAB using temperature, GHI, wind speed data, and technical data from manufacturers of these generators.

## CRediT authorship contribution statement

**Danilo P. e Silva:** Conceptualization, Methodology, Investigation, Writing – original draft, Writing – review & editing. **José L. Félix Salles:** Conceptualization, Supervision, Resources, Writing – original draft, Writing – review & editing. **Jussara F. Fardin:** Resources, Project administration. **Maxsuel M. Rocha Pereira:** Data curation, Methodology, Software, Resources, Writing – review & editing. **Vinícius C. Ottz:** Data curation, Writing – original draft, Writing – review & editing. **Flavio B.B. da Silva:** Funding acquisition. **Eduardo G. Pignaton:** Funding acquisition.

## Declaration of Competing Interest

The authors declare that they have no known competing financial interests or personal relationships that could have appeared to influence the work reported in this paper.

## Footnotes

1 The Global Horizontal Irradiance used in the manuscript is the total amount of shortwave radiation received by a surface horizontal 1,5 meters above to the ground.

2 The unit is wrong in the original .csv file. The correct unit is degrees.
